# Working with Policy and Regulatory Factors to Implement Universal Design in the Built Environment: The Australian Experience

**DOI:** 10.3390/ijerph120708157

**Published:** 2015-07-15

**Authors:** Helen Larkin, Danielle Hitch, Valerie Watchorn, Susan Ang

**Affiliations:** 1 School of Health and Social Development, Deakin University, Geelong Waterfront Campus, Locked Bag 20001, Geelong 3220, Australia; E-Mails: helen.larkin@deakin.edu.au (H.L.); valerie.watchorn@deakin.edu.au (V.W.); 2 School of Architecture and Built Environment, Deakin University, Geelong Waterfront Campus, Locked Bag 20001, Geelong 3220, Australia; E-Mail: susan.ang@deakin.edu.au

**Keywords:** universal design, occupational therapy, architecture, policy, built environment

## Abstract

Built environments that are usable by all provide opportunities for engagement in meaningful occupations. However, enabling them in day to day design processes and practice is problematic for relevant professions. The purpose of this phenomenological study was to gain greater understanding of the policy and regulatory influences that promote or hinder the uptake of universal design in built environments, to inform better future design. Focus groups or telephone interviews were undertaken with 28 key building industry and disability stakeholders in Australia. Four themes were identified: the difficulties of definition; the push or pull of regulations and policy; the role of formal standards; and, shifting the focus of design thinking. The findings highlight the complexity of working within policy and regulatory contexts when implementing universal design. Occupational therapists working with colleagues from other professions must be aware of these influences, and develop the skills to work with them for successful practice.

## 1. Introduction

The design of built environments is critically important to promoting engagement in meaningful occupations, and the capacity of all people, regardless of ability, to participate within their communities [1]. Research has confirmed a relationship between built environments, behavior, and health [2,3,4], however the links between health, architecture and design are not as well known. The link between participation and health and wellbeing is clearly established within occupational therapy [5,6], and the core business of many occupational therapists includes working with individuals and families to modify home and other environments within the content of health care services. However, Parnell and Wilding [7] recommend the profession look beyond these traditional practices, as “occupational therapists have something unique and important to offer those who plan and develop our shared built environments” (p. 345). They challenge occupational therapists to embrace and address more contemporary social and political issues as a means to advocate for occupational justice and inclusion for all. Occupational therapists in Australia are increasingly entering the multi-disciplinary area of the design of public built environments and spaces, often in the role of access consultant [8]. Access consultants are specialist professionals who conduct access audits, appraisals, research, and evaluation projects, consultations, advisory and training services around universal design in the built environment. These developments correspond with increasing recent interest in the principles of universal design, and their potential role in ensuring that public, and increasingly private, spaces and places facilitate and promote participation for all.

### 1.1. The concept of Universal Design 

Universal design refers to the creation of objects, resources, and built environments that can be used by the entire population, without adaptation or stigma, throughout their lifespan [9]. Originating from architecture in the USA in the early 1990s [10], the principles of universal design have become prevalent in diverse disciplines including occupational therapy [8], engineering [11], and architecture [10]. Universal design “seeks to eliminate discrimination by design and support full social participation for all members of society” ([12], p.165). The seven principles of universal design (see Table 1) were originally developed at North Carolina State University [13]. While there is some variability in the terms used to describe such non-discriminatory planning and design processes for built environments, (including *inclusive design, visitability, barrier free design,* and *design for all)*, universal design continues to be the most prevalent [8].

**Table 1:** The Principles of Universal Design (^©^1997 NC State University, The Center for Universal Design).

Principle	Descriptor
(1) Equitable use	The design is useful and marketable to people with diverse abilities
(2) Flexibility in use	The design accommodates a wide range of individual preferences and abilities
(3) Simple and intuitive use	Use of the design is easy to understand, regardless of the user’s experience, knowledge, language skills, or current concentration level
(4) Perceptible information	The design communicates necessary information effectively to the user, regardless of ambient conditions or the user’s sensory abilities
(5) Tolerance for error	The design minimises hazards and the adverse consequences of accidental or unintended actions
(6) Low physical effort	The design can be used efficiently and comfortably and with minimum fatigue

One of the earliest references to universal design in Australia arose out of the 2000 Summer Olympics in Sydney, dubbed “The Accessible Games” [14]. It has since been applied in settings as diverse as tourism [15], new homes [16] and aged care [17]. While the focus of universal design often centres on the needs of people with disability, Imrie and Luck [18] argue that much of the designed environment is inattentive to the needs of many people, and Lid [19] emphasizes that universal design is not about catering for the needs of one specific group of people. Indeed, Bringholf [20] argues that automatically linking universal design with the disability paradigm hinders progress towards the design of environments that are truly universal and inclusive in nature, by characterizing it as a “marginal” issue. 

While research into the uptake of universal design in Australian built environments is relatively sparse, the principles have been adopted as good practice by a range of organizations including AusAID [21], Disability Services Commission [22], Queensland Department of Public Works [23], Office of the Victorian Government Architect [24], and local planning authorities [25].

### 1.2. Legislation, Policy, and Regulations in Australia 

In 2006, the United Nations adopted the Convention on the Rights of Persons with Disabilities [26], which was ratified by Australia in July 2008. Accessibility is one of the founding principles of the Convention, and Article 9 explicitly states that signatories will take appropriate measures to ensure equal access to the physical environment in both urban and rural areas. Australia also acceded to the Optional Protocol, which enables individuals and groups to make complaints to the United Nations, once all domestic remedies have been exhausted, if they believe Australia is in breach of the Convention [27]. 

With Australia’s ratification of the United Nations Convention of Persons with Disabilities, the Council of Australian Governments established the National Disability Strategy [28] to build on the Conventions principles and ensure these principles were “incorporated into policies and programs affecting people with disability, their families and carers (and to) contribute to Australia’s reporting responsibilities under the Convention.” (p. 9). The Strategy has as the first of its six policy areas, a focus on “inclusive and accessible communities” (p. 10) and specifically highlights the need for inclusive and accessible buildings, housing, and other public spaces and places. Furthermore, the Strategy specifies that “the policies and practices developed by governments under the Strategy, including in the mainstream, will reflect and reinforce” (p. 23) a number of approaches including the need for a universal approach where “products, services, environments, and communities are accessible and usable by all people to the greatest extent possible without the need for specialized modification” (p. 23). The Strategy is significant in its use of the term “universal design” and also recognizes that universally designed built environments are beneficial for all members of the community. The Strategy is a significant shift towards a policy of moving beyond mere accessibility standards and a limited focus on the needs of people with a disability.

Under Australian legislation, the major Federal law relating to universal design of built environments is the Disability Discrimination Act. 1992 (Canberra, Commonwealth of Australia.). This Act stipulates that a person may not be discriminated against by denying them access to or use of public premises, unless the premises are existing and the provision of accessibility would impose ‘unjustifiable hardship’ on the person responsible for providing the accessibility. Thus, accessibility continues to be reinforced within the disability discourse that does not allow for a broader approach to addressing the barriers to health and well-being imposed by poor building design.

For many years, Australia has had well-established Standards that determine the requirements for disability access for public premises [29]. In order to further strengthen their regulatory significance, the Australian government in 2010 committed to codifying individual rights to access public premises within each State’s Building Code, and this was applied to all new buildings after May 2011 [30]. 

In 2009, the Disability Investment Group [31] called for urgent action to make mandatory, national building standards designed to ensure the increased availability of universally designed private housing to accommodate an anticipated rapid increase in the age of the population and number of Australians with a disability over the next 40 years. As a result, they recommended that all new homes have a minimum set of specified access features, as shown in Table 2. 

**Table 2:** Minimum Access Requirements Recommended for Australian Private Housing.

Accessibility Recommendations for Australian Housing
A continuous accessible path of travel from a parking area or allotment boundary and a level entry into the home;
A bathroom on the ground floor with reinforced walls, to allow for future adaptation and a step-free shower recess;
External and internal doorways with a minimum 850mm width;
Corridors on entry level with 1000 mm width;
Space on the ground floor capable of use as a bedroom or living area; and,
A kitchen area capable of adaptation to provide sufficient turning space between benches.

These visitability requirements were taken on board by Livable Housing Australia [32], and nationally agreed guidelines for new homes were developed the Kirribilli Dialogue on Universal Housing Design. The focus of these guidelines is on housing that addresses the needs of a broad percentage of the community to “better meet the changing needs of occupants over their lifetimes” ([32], p. 5). Livable Housing Australia argue that “Livability works for pregnant mums, young families with kids and people with sporting or traumatic injuries, as well as seniors, Australians with disability and their families. Livability is an investment that makes both economic and social sense. It also offers peace of mind.” (p. 5). In partnership with key building industry stakeholders, aspirational targets were set for a percentage of new housing to incorporate these livable housing requirements. However, it appears that the 2020 targets are unlikely to be met and that this aspirational and voluntary (as opposed to a mandatory) approach, has not been sufficient to bring about change [33]. Encouragingly however, some isolated local council planning authorities have taken up the challenge of increasing the supply of private housing incorporating livable housing features [34].

Professions working in the built environment in Australia therefore have both political and regulatory frameworks to support their efforts to enact universal design. This study sought to investigate the experiences of key industry stakeholders, including occupational therapists, architects, and others, around the uptake of universal design for Australian built environments. As part of a larger study, it addresses a substantial gap in the literature around inter-professional perspectives of the legislative and regulatory context that influences the design of built environments.

## 2. Experimental Section

### 2.1. Study Aims 

This study informed a larger study of inter-professional education, that introduced universal design practice into the curricula of undergraduate architecture and occupational therapy students at a regional campus of a large Australian university [8,35,36]. The aim of this element of the overall study was to investigate the views and perspectives of key industry stakeholders, to identify factors that promoted or hindered universal design in practice so they could be addressed in the developing curriculum. 

### 2.2. Methods 

The study was approved by the Human Ethics Advisory Group, Faculty of Health, Medicine, Nursing and Behavioural Sciences, Deakin University, Australia. It used a phenomenological approach with open-ended questions designed to explore the everyday experiences and views of participants about key issues and industry practices [8,37].

#### 2.2.1. Participants

A total of 76 people were invited to participate, with 28 individuals subsequently taking part in a focus group (n = 16) or individual telephone interviews (n = 12). Focus group participants were recruited from two major metropolitan centres in the same State, while telephone interview participants were located across three mainland Australian States. Participants were initially recruited by judgement sampling, as members of the project steering committee nominated informants known to have expertise and experience in universal design and built environments. Snowball sampling also occurred, as participants recommended other key informants who may be interested in participating. Recruitment continued until the point at which saturation of the data occurred [38]. 

Of the 28 participants, 75% (n = 21) were women and 25% (n = 7) were men. Almost half (46%) were aged 45–54 years, with a further 21% aged 35–44 years and 14% aged 55–64 years. Participants were all experienced in terms of building design and accessibility, reporting a mean of 20.22 years working in the field of environmental accessibility. In terms of professional background or role, 34% (n = 9) were occupational therapists, 11% (n = 3) were architects and 18% (n = 5) identified as service managers representing agencies that supported and advocated for people with disabilities. The background of remaining participants included law, higher education and other health professional roles. Across the sample, 29% (n = 8) also identified as access consultants. Several participants (33%, n = 4) also identified as people with disabilities, and brought both personal and professional experiences of environmental and universal design to the study.

#### 2.2.2. Data Collection

An introductory email was sent to key informants inviting their participation, and a plain language statement was also provided at that time. Informed consent was confirmed in writing prior to participation, with a brief questionnaire used to collect demographic information prior to commencement of the focus group or telephone interview. Three focus groups were held with a facilitator and observer/notetaker in attendance, and 12 telephone interviews were conducted by a single interviewer at a time convenient to the participant. All focus groups and telephone interviews were digitally recorded, and transcribed verbatim [33]. 

#### 2.2.3. Data Analysis

All transcripts were read several times to ensure that the researchers were immersed in the data, and thematic analysis was undertaken with relevant codes assigned on a line-by-line basis. A list of generated codes was developed from the data contained in all transcripts, and these were later clustered into themes. All four aspects of trustworthiness defined by the Rosalind Franklin Qualitative Research Appraisal Instrument (RF-QRA) [39] were addressed in this study. Credibility (internal validity) was enhanced by the recruitment of informants from a range of professional backgrounds, ensuring that multiple sources and perspectives were accounted for. While generalizability (external validity) is not the aim of qualitative research, the sample included representatives which mirrors the profile of the population of key informants and stakeholders in the field of universal design in Australia. Dependability (reliability) was promoted by peer examination of the final coding list and thematic structure, and confirmability was enhanced by the presence of an observer / note taker at the focus groups, and regular review meetings of all members of the research team.

## 3. Results and Discussion

Four themes that emerged from the data related to the policy, political and regulatory factors that influence the uptake of universal design in Australia. These were: difficulties of definition; the push and pull of policy and regulatory influences; the role of formal minimum standards; and, shifting the focus of design thinking.

### 3.1. Difficulties of Definition

The lack of a commonly agreed terminology was identified as a barrier to progressing design practice that includes the needs of the broadest range of people and embraces diversity of abilities and backgrounds.

*If you say inclusive, it does make you think ‘who am I excluding if I don’t have inclusive?’ Whereas universal sounds more like ‘let’s make it all the same’. So I like inclusive because it’s an active word.*

*I like universal because it’s more egalitarian. Universal implies to me that everybody can do it.*

*Inclusive implies someone’s doing me a favour, that they’re including me, whereas in universal, it appears that everyone has equal access.*

*When we use the word inclusive, we cannot explain the word inclusive without specifying who is excluded, who are ‘the others’ and that is why the whole thing has a language difficulty.*

While there was disagreement in relation to choice of preferred terms, there was a shared understanding about what characterized this sort of design thinking.

*Inclusive design that suites people of all abilities….this is a whole community approach, this is not just about making our hospitals accessible for people with disabilities.*

*Something that includes everybody, so design doesn’t label people, that is just there for everyone to use …design for everyone, to include all abilities, ages.*

*All people, accessible to all people. People from different background, cultures, physical abilities.*

While participants felt that design needed to be targeted to the broadest range of people, difficulties were noted in terms of the poor application or operationalization of these principles.

*It’s not just about getting in the front door; it’s what you do once you’re inside. It’s getting around places.*

*Sometimes, you can have a great facility that’s very accessible but you get there and the staff or the people who run the organization or the facility have no idea about engaging or supporting or assisting people with disability, therefore it’s of minimal use.*

*I realized that there was this flawed assumption that if you design for somebody in a wheelchair, that’s the bottom line, that’s the worst case scenario so you will cover everybody … disability does not equal wheelchair.*

There was consensus with participants viewing inclusive or universal design being about a lifespan approach.

*I think about go to woe, from pushing a pram to being a very old person and having a design that suits everyone.*

*We’re including everybody who at some time in their life might be excluded.*

### 3.2. The Push and Pull of Policy and Regulatory Influences

The role of political and regulatory influences in Australia drew mixed responses from participants. Some felt that they needed to take the lead, while others felt responsibility for its implementation lies elsewhere.

*It needs to come from the government. All the building commissions, they need to be given those directives.*

*I think we need political will because we can bat on about it for decades but until a politician has a person in their family with a disability; it’s not put on the table.*

*People have the debate of will it just come when the consumers demand it, will it come when governments require it, will it come when architects are inspired to design elegantly for people with disabilities or the broader community and I’m not sure. ... I think that it’s clearly going to come from all of those things working in concert to create the demand and respond to the community’s call for inclusion.*

For some participants, the development of relevant legislation in recent decades was seen as a positive development from the point of view of raising awareness.

*More and more legislation we have. As its progressed, more and more people in the industry have become more and more aware of it.*

*I think government of all levels, local, state and federally are certainly more aware today, so that’s a plus.*

However, others were quite scathing in their opinions about legislative support, and particularly about the length of time it takes for legislation to be passed and come into law.

*When DDA first came into place, it went off with a bang and the government has delayed. It’s taken them 15 years to get the Access to Premises Standards up and running. I think they’ve deliberately done that so that all the fighters out there advocating just give up or go away, and that’s what they want.*

*It always seems to be lagging slightly behind current thinking and uses a sledgehammer to crack a nut.*

*Legislation wise it’s been a disaster. The Disability Discrimination Act was enacted in 1993. We’ve [only] got a transport standard and now we’ve got a premises standard. It’s just diabolically bad … I think the legislation’s largely failed us and is continuing to fail us.*

### 3.3. The Role of Formal Minimum Standards

Participants also expressed frustration with formal standards such as the building code and new premises standard. The most prevalent criticism was that these standards promote a “tick the box” and “lowest common denominator” approach to universal design which is more about meeting criteria than meeting consumer need.

*I think unfortunately when it comes down to it, our clients’ ‘bottom line’ is they just want to meet Building Code of Australia requirements, of what they have to do, and that’s quite minimal. They’re not really concerned about universal design, they’re concerned about meeting the regulations … That’s what we have to do to comply with, to get our tick of approval from the building surveyor.*

*I think regulations are dangerous because people think that’s all they’ve got to do, and they’re just the minimum, and we’ve got to encourage people to go past it. Any architect who’s worth his sod should see those as a minimum standard, and then know in special circumstances you do more over and above.*

Related to this was the mistaken idea that compliance with the regulations meant that you had “done” universal design; that these documents represented the totality of the subject.

*The raft of regulations, whether it’s the Australian standards or the Building Code of Australia or any of the explanatory and guide notes are, in my view, just that. They are guide documents. They will tell you what you can’t do, but they very seldom tell you what you can do … It’s possible to design a building that is absolutely and 100% compliant in the regulatory sense of the word but is absolutely inaccessible in every other sense of the word.*

*I think one of the difficulties is there are some building standards, but people with disabilities will tell us that many of the barriers relate to more than just the physical getting in. So I think the building standards, you’ve got something to hang your hat on, and you’ve got the human rights charter for people with disabilities around their rights, but if they’re ignored in a shop which is often the complaint we get … that doesn’t mean inclusion and it doesn’t mean participation.*

For some participants, the main problem was that standards that do exist aren’t being implemented even at the minimum level.

*You work pretty hard to try and get small improvements … so much gets approved and passed that is not correct, it doesn’t meet current building codes and standards all the time.*

Participants also questioned the role of standards when it came to the enforcement of consequences. While some felt their power was weak as enforcement rarely happens, others saw the penalties for non-compliance as punitive and counter-productive.

*There aren’t a lot of consequences for not doing it. So sometimes it’s like, what’s going to happen if I don’t do it? Not much! … so you can use the carrot or the stick can’t you, so you can make this great for people or you can, how dare we restrain/restrict people with our non-inclusive designs … There are fines that are going to mean something, it’s not just a slap on the wrist, there’s a disincentive not to do it.*

*People provide access because the law requires them to, not because they understand the reasons for it.*

*It didn’t go down well with some business owners all that well because they saw it as being a tedious money-making scheme by the government and found it really quite annoying.*

### 3.4. Shifting the Focus of Design Thinking 

Participants were in agreement that if we are to progress the move towards more inclusive and accessible built environments, that a change in the way of thinking is required and perhaps a different angle to the marketing of such design.

*Architects have now been talking about sustainability and that’s kind of accepted as part of a philosophy, whereas universal design is not quite there yet.*

*Universal design thinking is not actually that far removed from ecologically sustainable design principles in that we’re trying to move people forward into understanding the materials we use affect our health and they do affect our wellbeing.*

*I think the challenge is to make it sexy … and there’s an absence of marketing around this to make accessibility sexy … this area isn’t very sexy at times. When you talk about inclusive design or universal design, it’s tagged with disability and disability isn’t sexy.*

*Universal design is still something that is slowly creeping in, but it hasn’t really made an impact here in Australia. We’ve tried in our company to include the word in our fee proposals and trying to filter that through into our consultancy advices and design reviews, but that’s been a slow process because we have to start re-educating our clients to what this actually means.*

*Having some marketing campaigns that focus on universal design, and evaluating the effectiveness of that in relation to changing the practice of builders.*

### 3.5. Discussion 

Findings from this study suggest some key political and regulatory tensions and barriers to the implementation of universal design of built environments in Australia. The lack of agreement regarding the most preferred term, whether it be universal, inclusive, or livable was seen as problematic to consistent application or enactment. However, there was also a view that the design intent and processes were more important than the terms used to describe them. Participants thought that placing inclusion at the centre of the design process was a hallmark of generally ‘good design’, as was incorporating meaningful collaboration with users and adopting a lifespan approach. These messages are consistent with the most recent literature, which emphasizes that the enactment of universal design extends well beyond the disability discourse. As highlighted by Steinfeld, Maisel, and Levine [10], there is a growing recognition that (regardless of the terminology used) built environments need to accommodate the needs of entire populations. This study also supports the views of those who’ve warned of the dangers of isolating universal design in the disability paradigm [20] and who recommend we spend less time and energy on the words used and more time on the design intent [33,40]. The focus of discourse around universal design is increasingly focusing on process rather than end product.

However, the focus on definitions and terms reflects a reality of the current policy and regulatory framework around universal design, particularly where specific standards are introduced. This study has highlighted an important distinction between visitability/accessibility and true inclusion. As stated by the participants, universal design needs to go way beyond simply allowing people to get into built environments, to encompass the occupations they wish to participate in within those environments. Other people play a key role in our interaction with the built environment, and the participants commented on the need for appropriate education of staff in universally designed facilities as poor attitudes can negate positive elements of good physical design. If the focus of universal design stops at getting people into the space, their participation in roles other than “visitor” may not be enabled. Too often public spaces and places are well designed, but the “back of house” working spaces are not conducive to people with mobility or other impairments. In these cases, anyone may visit the environment, but not everyone could work there. With this in mind, it would appear that the term “visitable” does nothing to enhance a broader design agenda that embraces the inclusion of all members of the community in social and work-related roles. The recent emergence of “livable” in relation to private housing [32] may offer a more appropriate alternative, and a similarly appropriate term may be found in the future for public spaces and places.

The study findings support the view that despite improved regulatory standards, builders, developers and others continue to apply a “tick the box” approach to universal design, where compliance takes precedence over potentially adding market value. Participants suggested a need to re-vision the branding of universal design in a desirable and marketable way, with one participant suggesting making use of the momentum around sustainability as a key design imperative for the future. Some sources [10,23] have indicated that universal design is increasingly being viewed as an essential element of sustainable design, as where “the occupant can move around more easily, feel safer, save money and use resources like energy and water more efficiently … that will meet a family’s needs through all stages of their lives” ([23], p. 2). 

Livable Housing Australia worked with building industry stakeholders as champions of change in their development of the 2013 aspirational guidelines for the Australian housing industry. However, some still expect that these voluntary codes will be unsuccessful in achieving the stated targets [41] noting that the lack of demand from consumers previously identified by Keates and Clarkson [42] continues today. In 2014, the Australian Network for Universal Housing Design and RI Australia [43] reported that “the housing industry has failed to show any sign of systemic transformation” (p. 1). If universal design could be seen as an essential element of sustainability, and incorporated into the corresponding legislated certification process, then perhaps true change can occur [10]. It seems likely that the design sector could learn from global public health initiatives (such as the anti-smoking lobby in Australia), and adopt a combination of legislation, taxation changes, education, smart design, and health services to spur the complex changes in attitudes and behaviours required to get universal design into regular practice. 

Our study reinforces other authors’ calls for further studies into the economic benefits of applying universal design, to counter the commonly held view that such practices are more expensive. The assumption that universal design adds costs does not take a sustainable, lifespan approach or consider future users of the building and the need to avoid excessive costs associated with retro-fitting of buildings at a later stage [8,16]. The participants in this study also emphasized the value of engaging user groups as early as possible in the design process, again highlighting that universal design is as much about the process as it is about the end product. While there is some acknowledgement as to the importance of this as a valuable principle, there is limited evidence or research into the manner in which this can best be undertaken.

The findings from this study provide new insights into the impact of legislative and regulatory context on universal design of the built environment in Australia. There are however, some limitations that impact on the generalizabilty of the findings. Not all areas and jurisdictions in Australia were represented, and the sample was dominated by two professions in particular (occupational therapists and architects). More broadly, the findings here are bound to their national context, and further research would be needed to test the validity of these themes in others countries. Despite these limitations, the study has been successful in gaining a greater understanding of the policy and regulatory influences that promote or hinder the uptake of universal design in built environments in Australia. The findings and resulting discussion have also highlighted some potential measures to improve future design, and support the broader uptake of universal design. 

## 4. Conclusions 

As argued by Hitch, Larkin, Watchorn, and Ang [8], good design benefits everyone and should not be seen merely for the paternalistic benefit of one section of the community. Such an approach not only continues to marginalize people with a range of health conditions and impairments, but turns its back on the opportunity for a broad range of people, regardless of ability, to benefit from good design (p. 381). However, translating good intentions in to tangible changes in practice is a very complex undertaking, even in the context of substantial political and regulatory supports. 

This study indicates that the major facilitators for enacting universal design in the built environment in the coming years will be a shift in focus away from disability, greater collaboration with diverse users during the design process and a diversification of strategies used to support the necessary changes. Occupational therapists are well placed to make a positive contribution in this field with their understanding of diversity and inclusion, and growing skills in advocacy and political practice. The profession’s willingness to take an interdisciplinary approach will also be crucial, as the solutions we seek will only come about from collective effort. The complexity and challenge associated with enabling these changes may seem overwhelming, but Stephen Hawking offers this perspective,

*In another 20 years from now, man will be able to live on the moon, and in another 40 years, man will be able to live on Mars. In the next century, we'll be able to cross the limits of the solar system and search for new worlds. But in the meantime, we really want to go to the supermarket, the movies, and to a restaurant.*

As occupational therapists, it is clearly our role to enable individuals to engage in meaningful occupations like shopping, leisure, and socializing. We must become skilled in working with complexity within political and policy frameworks to transfer our existing specialist knowledge and skills to advocacy around universal design in the built environment for the community as a whole. 
